# Supplementary material for: Quantifying global colonization pressures of alien vertebrates from wildlife trade
Source: Nat Commun. 2023 Nov 30;14:7914. doi: 10.1038/s41467-023-43754-6 (PMC10689770; doi:10.1038/s41467-023-43754-6)
Supplement: Supplementary file 1 — Supplementary Information [file 41467_2023_43754_MOESM1_ESM.pdf]

Quantifying Global Colonization Pressures of Alien Vertebrates from Wildlife Trade

Yiming Li, Tim M. Blackburn, Zexu Luo, Tianjian Song, Freyja Watters, Wenhao Li, Teng Deng, Zhenhua Luo, Yuanyi Li, Jiacong Du, Meiling Niu, Jun Zhang, Jinyu Zhang, Jiaxue Yang, and Siqi Wang

Supplementary Information

Supplementary Note 1. Abstract in Chinese

量化贸易外来陆生脊椎动物在全球的定居压力

摘要：全球野生动物活体贸易增加了外来物种的定居压力（即引入到一个区域的外来物种数），抬高了生物入侵的风险。然而，目前人们对贸易物种中有多少是外来物种，知之甚少。我们建立了活体陆生脊椎动物贸易全球数据库，并用它来调查贸易外来物种的丰富度，以及外来种建群丰富度的影响因素。我们发现，全球野生动物活体贸易包括 7780 种陆生脊椎动物（哺乳动物，鸟类，爬行动物和两栖动物），这些物种中，85.7%是外来种，12.2%的外来种已建立了野生种群。大量的外来种进口到进出口贸易量大，人口基数大和人均 GDP 高的国家。这些国家，以及一些岛屿国家，也是外来种种群建立的热点区域。定居压力和岛屿属性一致性地助长建群丰富度，而社会经济因素影响个别类群。政府部门有必要优先制定管理贸易动物释放或逃逸的政策法规，以保护全球的生物安全。

Fig S1-S7

Fig S1. Venn Diagram of species assembled from different data sources for four groups. The figure is created by the VennDiagram package in R (Venn Diagram in Supplementary Code 1). a, mammals; b, birds; c, reptiles; d, amphibians.

a, mammals

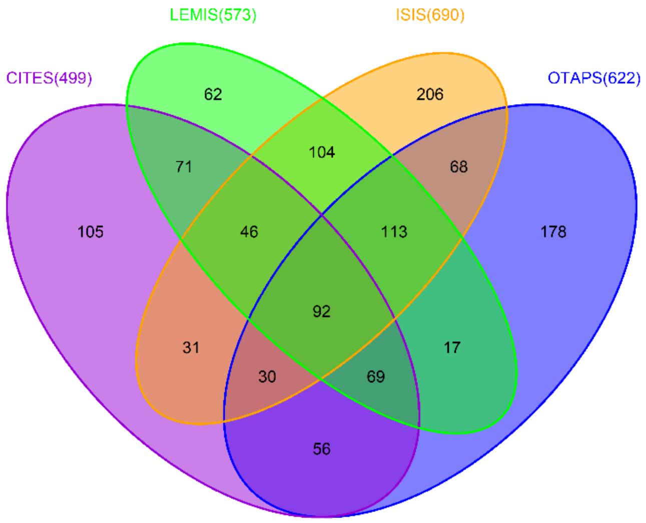

b, birds

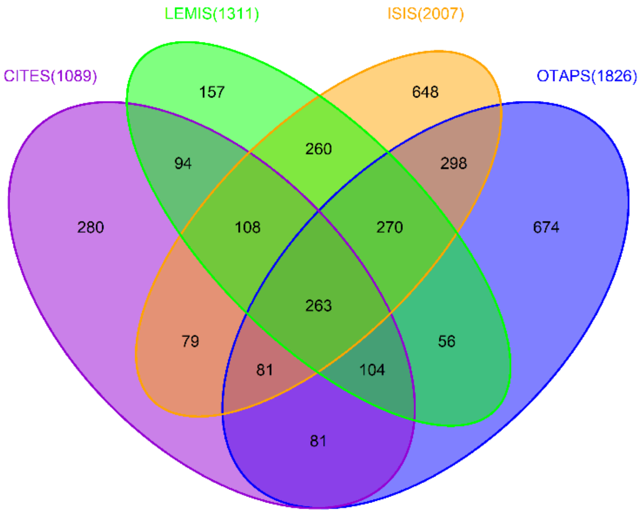

c, reptiles

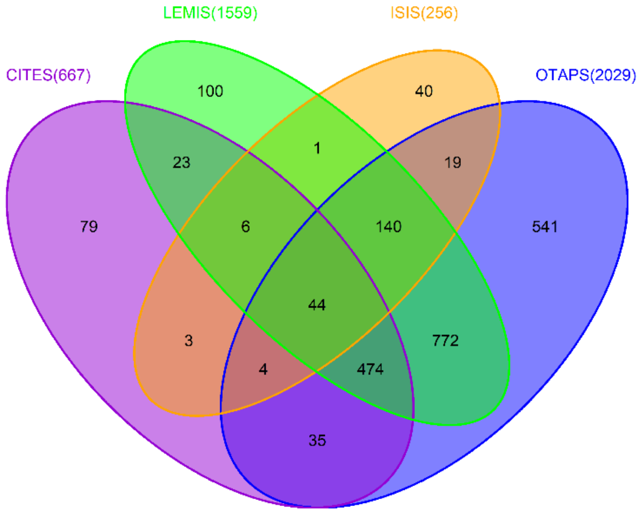

d, amphibians

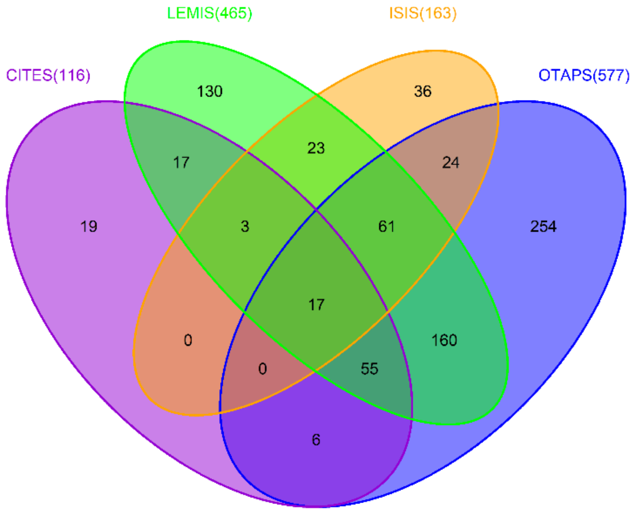

Fig S2. The geographical distribution of the numbers of alien vertebrate species in trade for four groups across countries and regions based on GLVTD (also see Supplementary Data 2). The figure is created by ArcGIS. The alternative versions of the figures are provided with e-i. a, mammals; b, birds; c, reptiles; d, amphibians; e, vertebrates with alternative version; f, mammals with alternative version; g, birds with alternative version; h, reptiles with alternative version; i, amphibians with alternative version.

a, mammals

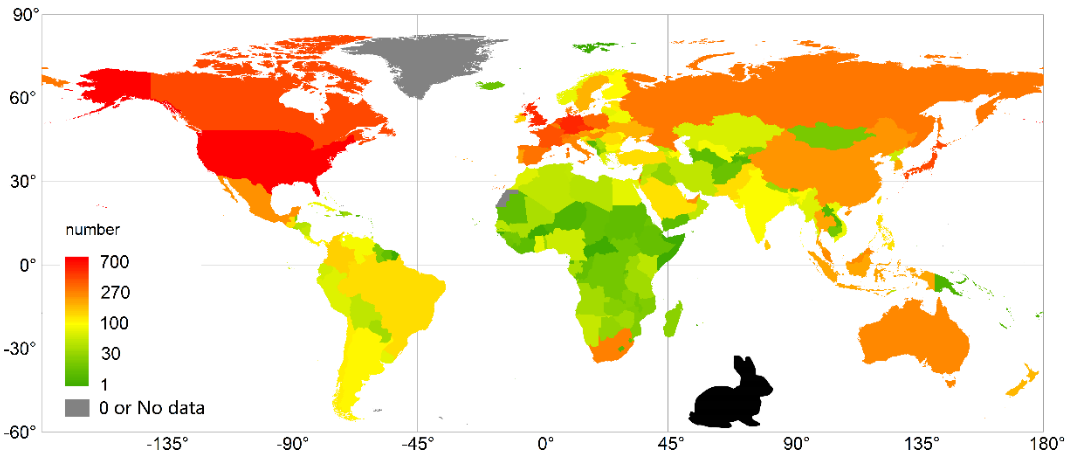

b, birds

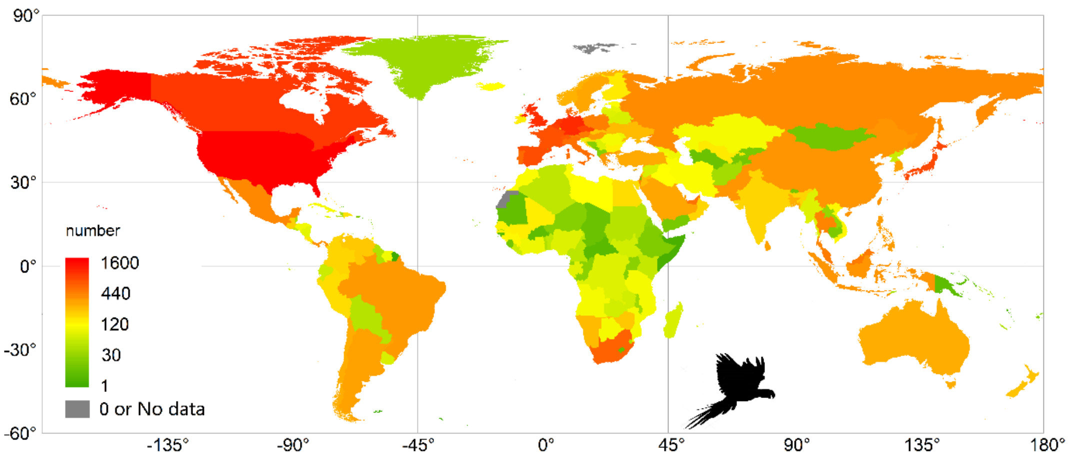

c, reptiles

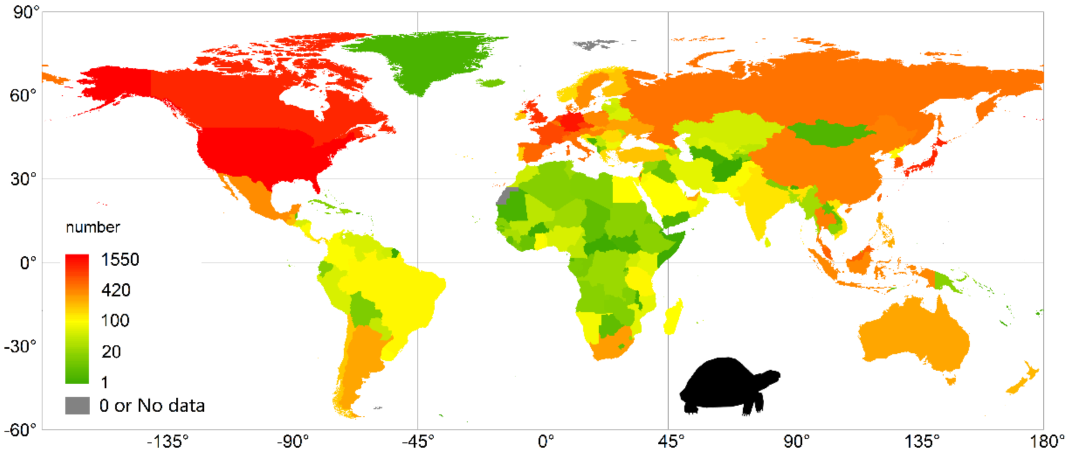

d, Amphibians

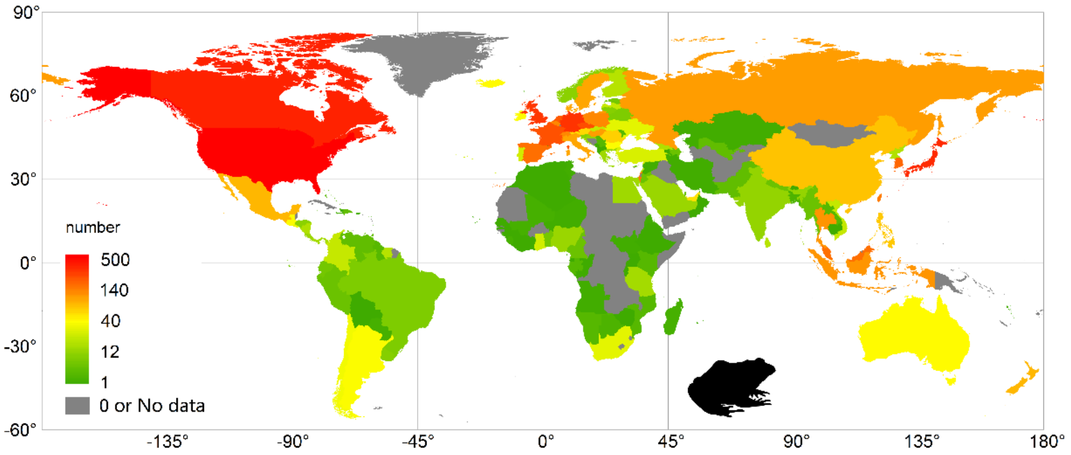

e, vertebrates with alternative version

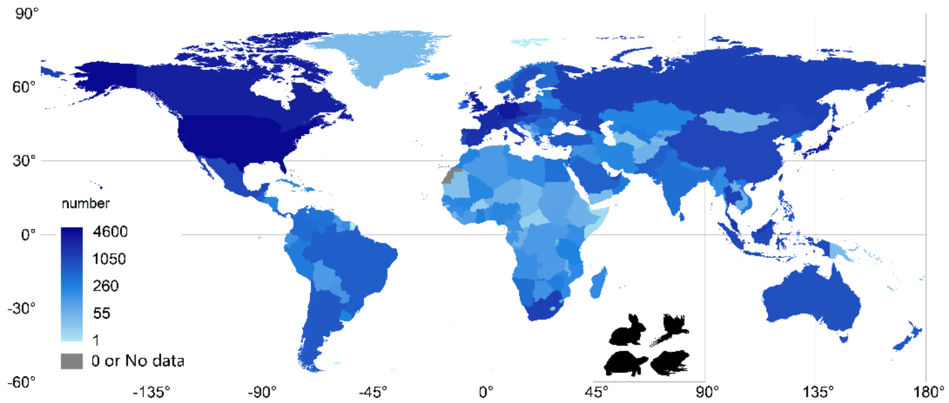

f, mammals with alternative version

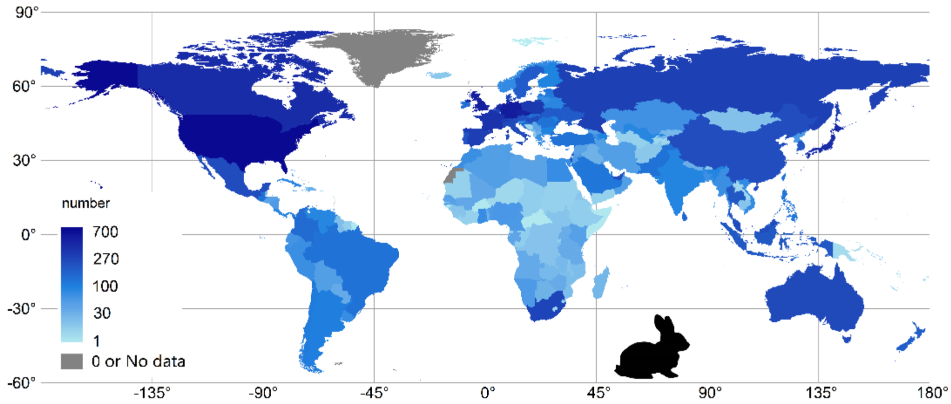

g, birds with alternative version

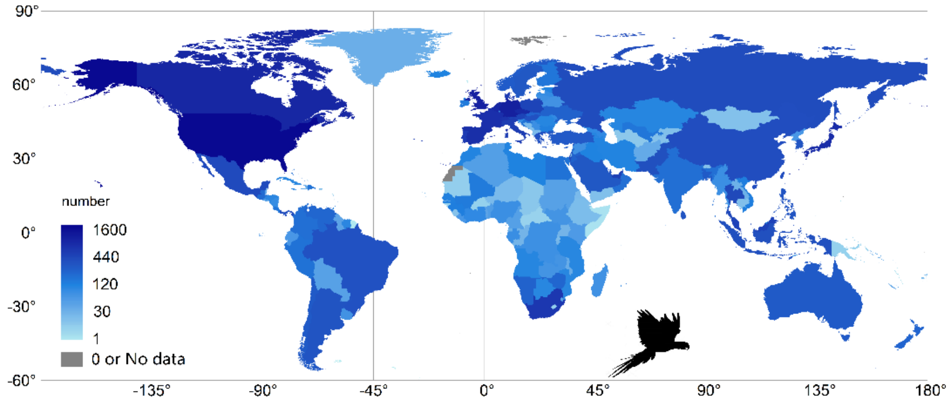

h, reptiles with alternative version

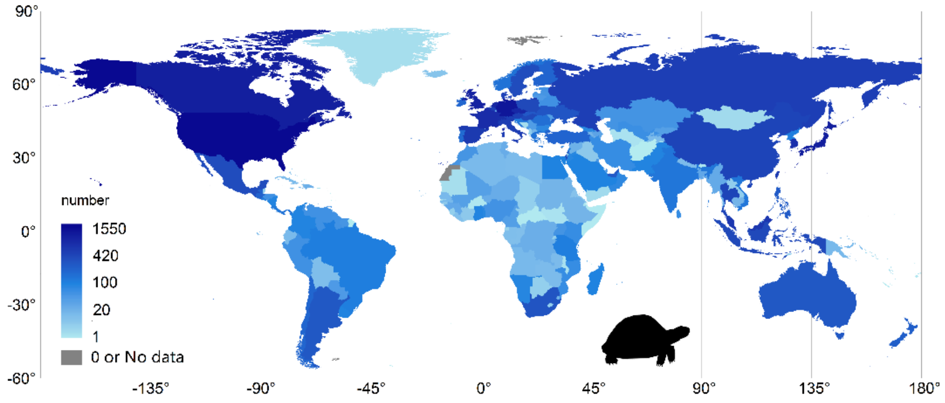

i, amphibians with alternative version

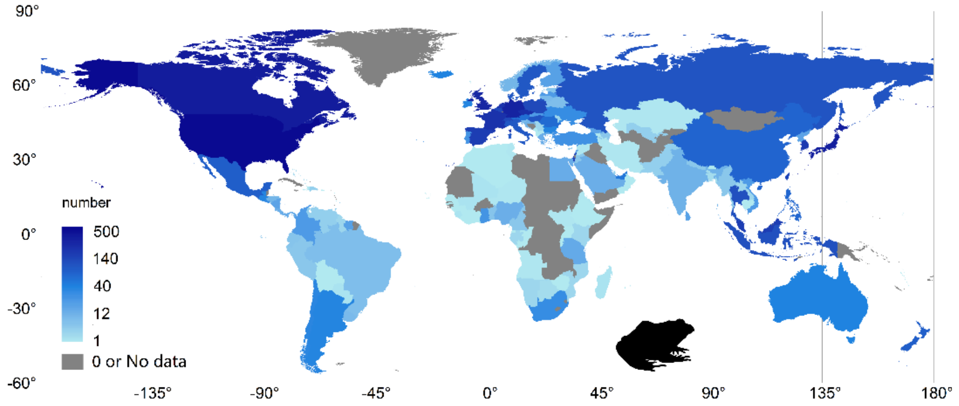

Fig S3. The geographical distribution of the numbers of alien vertebrate species in trade with established populations (i.e. established alien richness) for four groups across countries or regions based on GLVTD (also see Supplementary Data 4). The figure is created by ArcGIS. The alternative versions of the figures are provided with e-i. a, mammals; b, birds; c, reptiles; d, amphibians; e, vertebrates with alternative version; f, mammals with alternative version; g, birds with alternative version; h, reptiles with alternative version; i, amphibians with alternative version.

a, mammals

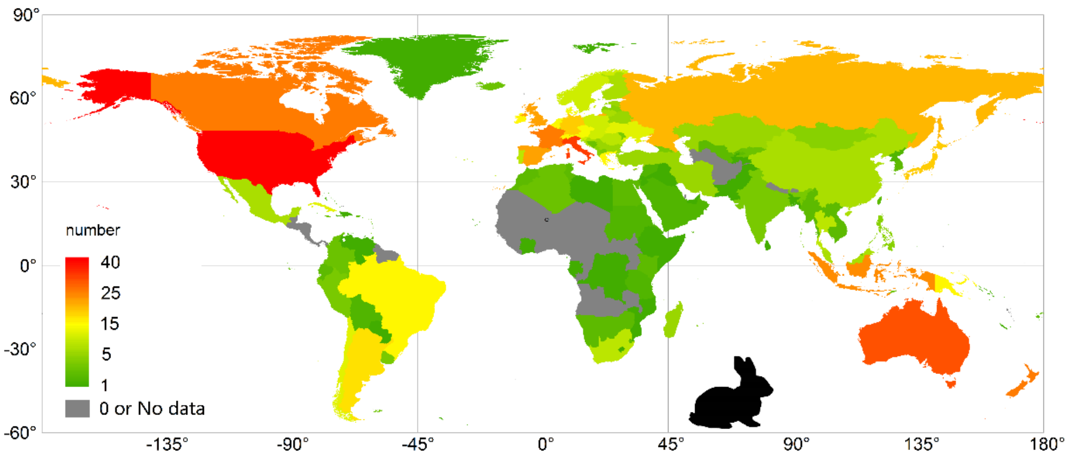

b, birds

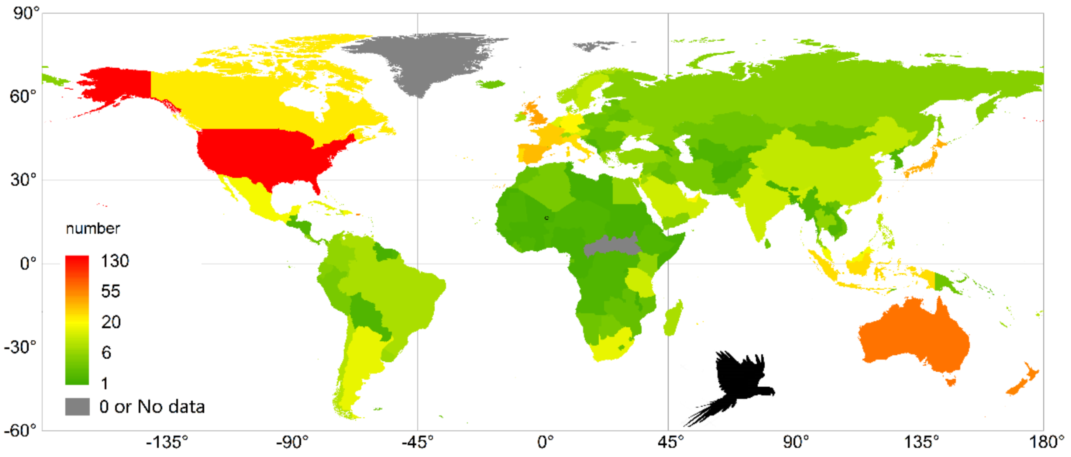

c, reptiles

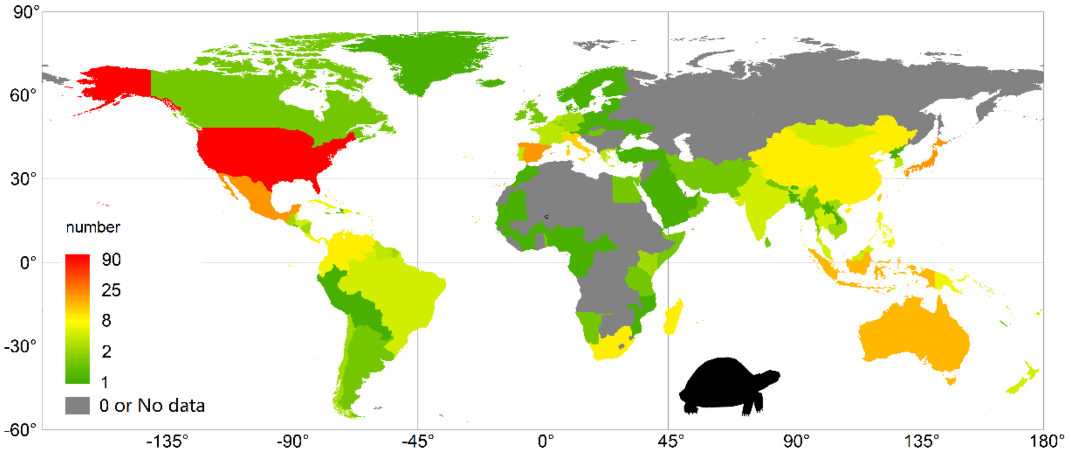

d, amphibians

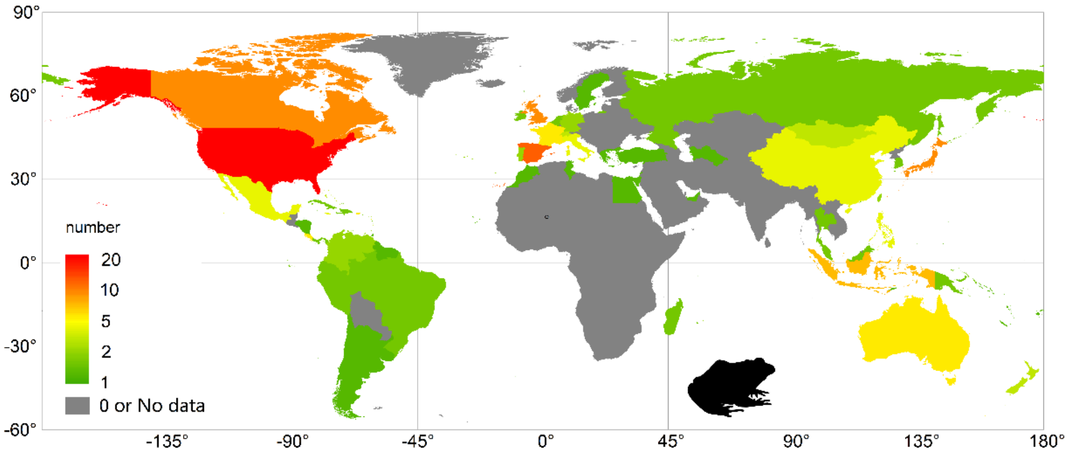

e, vertebrates with alternative version

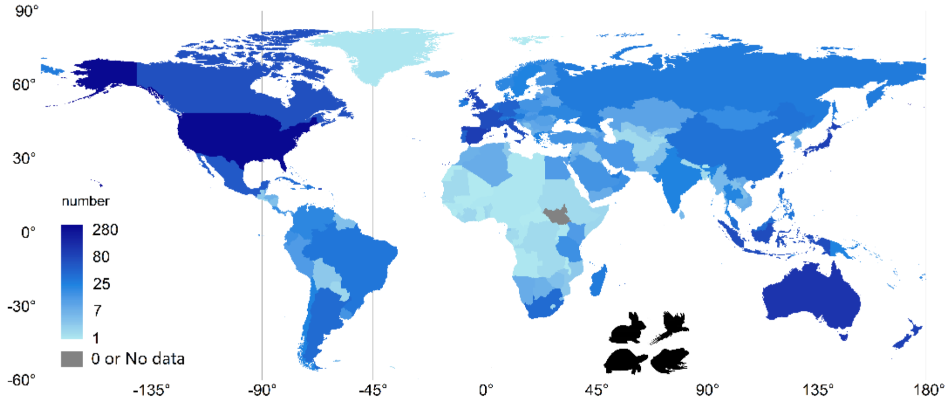

f, mammals with alternative version

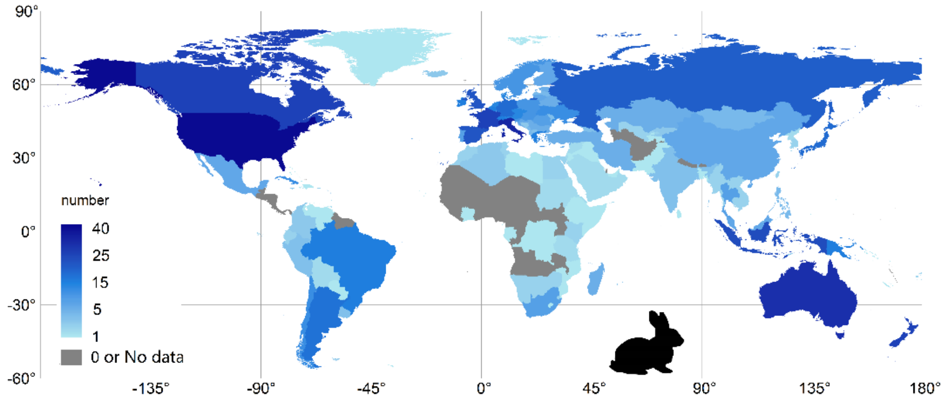

g, birds with alternative version

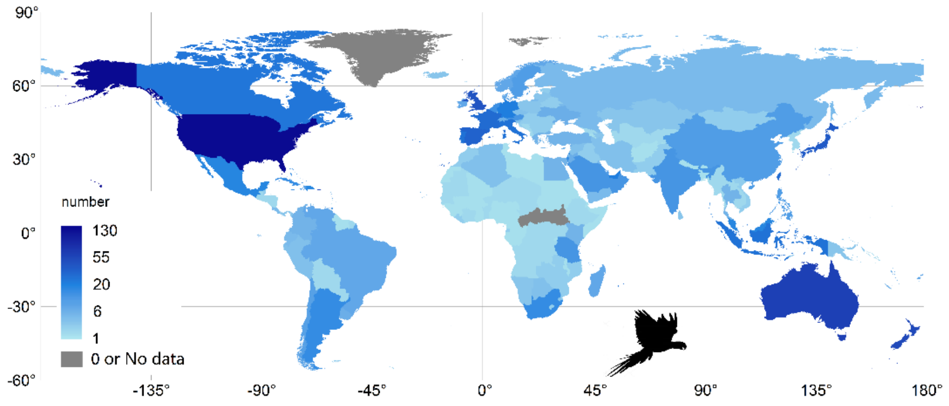

h, reptiles with alternative version

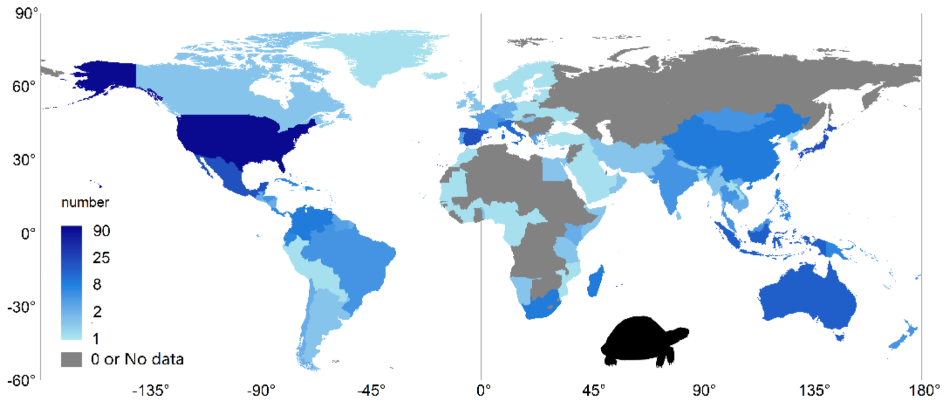

i, amphibians with alternative version.

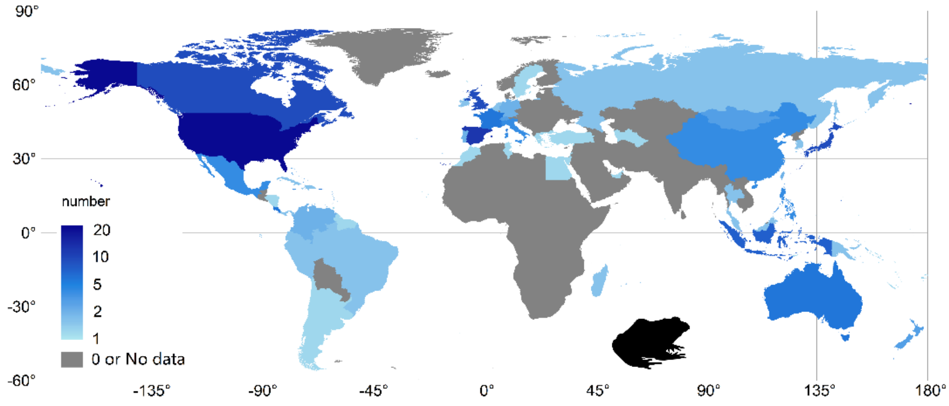

Fig S4. The boxplot of number of countries involved in trade between established and unestablished species across 193 countries for each taxon. Source data are provided as a Source Data file. The figure is created by Excel.

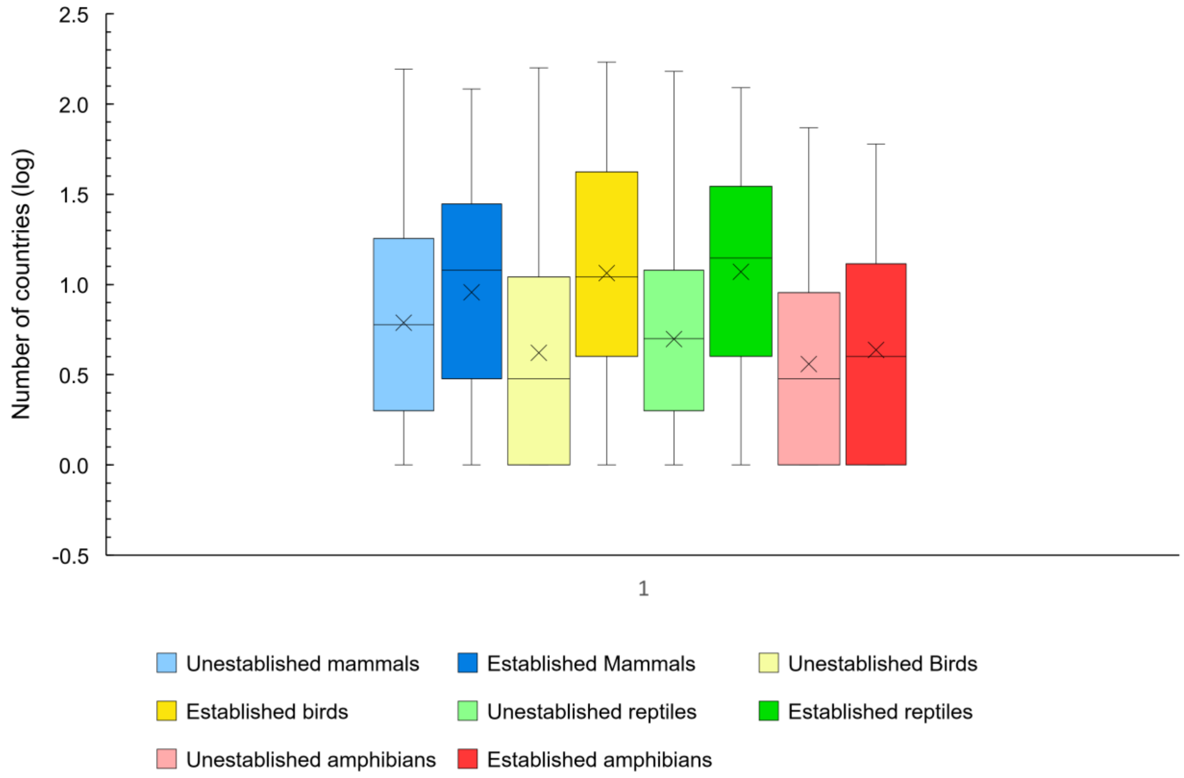

Fig S5. The boxplot of areas (km^2) involved in trade (summing the areas of countries with trade) between established and unestablished species across 193 countries for each taxon. Source data are provided as a Source Data file. The figure is created by Excel.

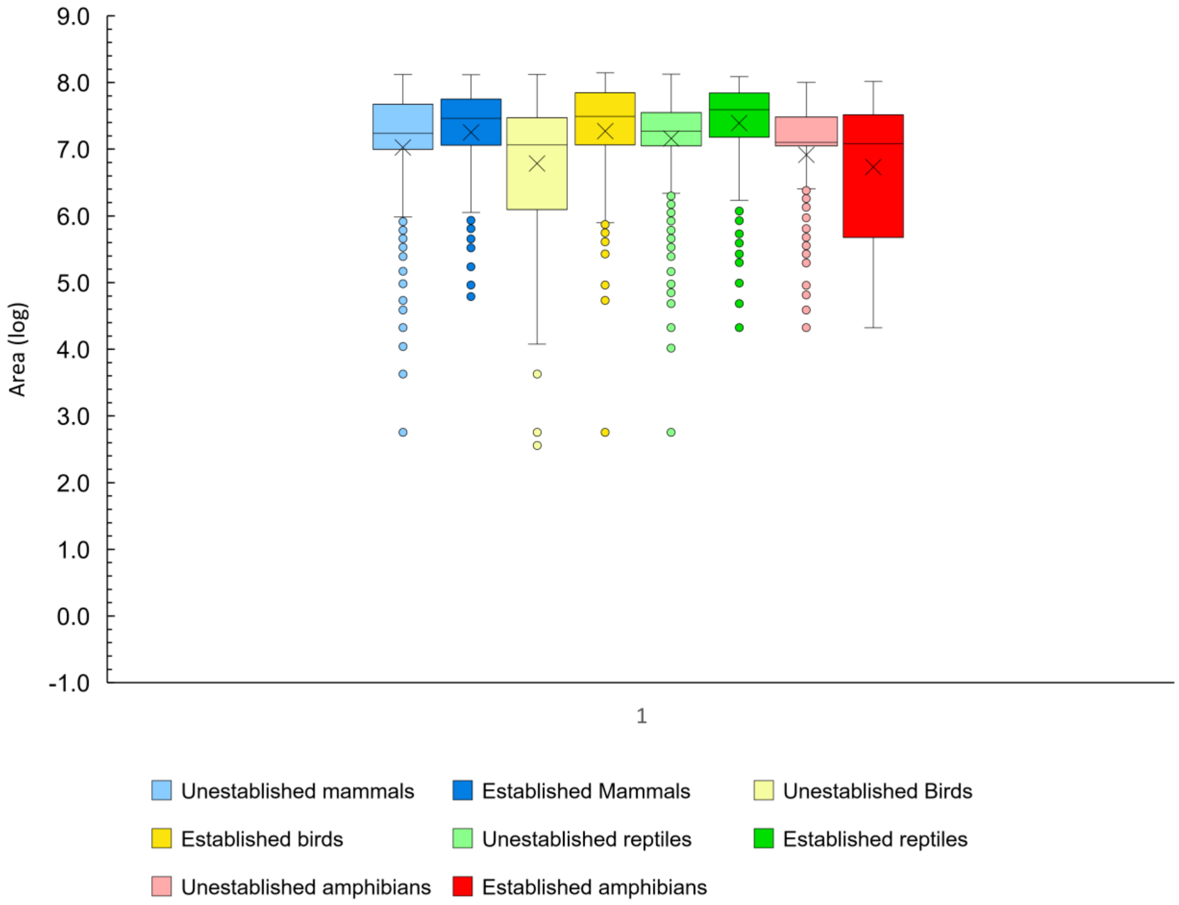

Fig S6. The global network of traded alien wildlife in term of species number for four taxonomic groups (see Fig 5 for details). The figure is created by the dplyr, circlize and reshape2 packages in R (Network analysis in Supplementary Code 1). a, mammals; b, birds; c, reptiles; d, amphibians

a, mammals

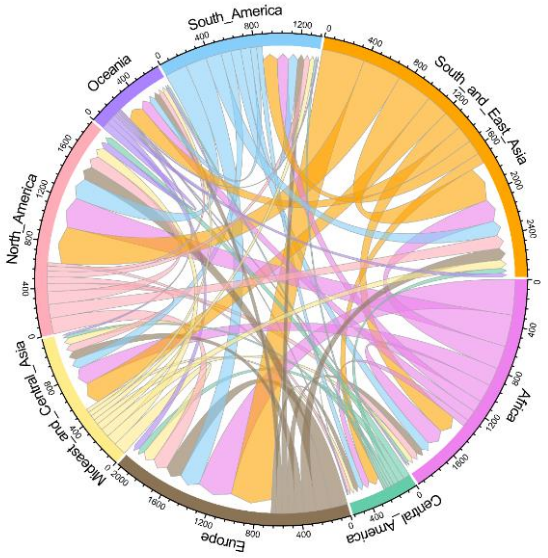

b, birds

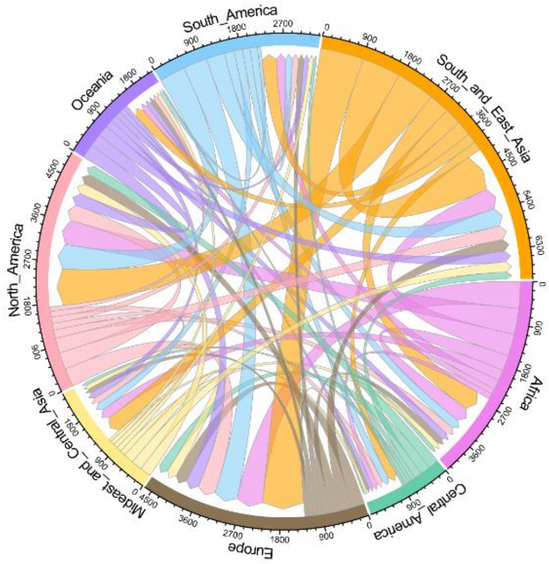

c, reptiles

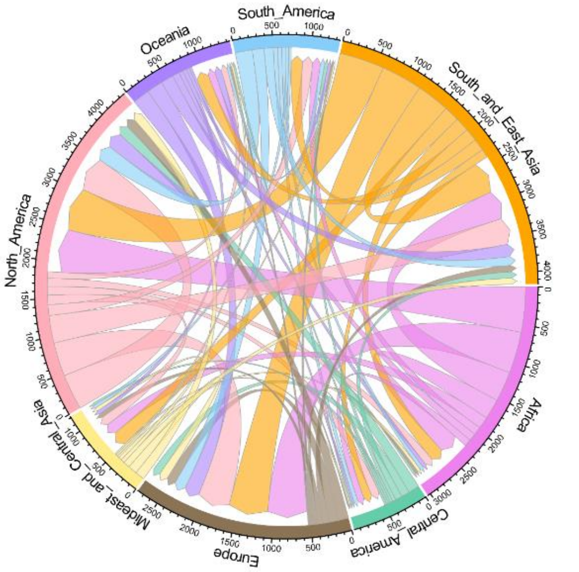

d, amphibians

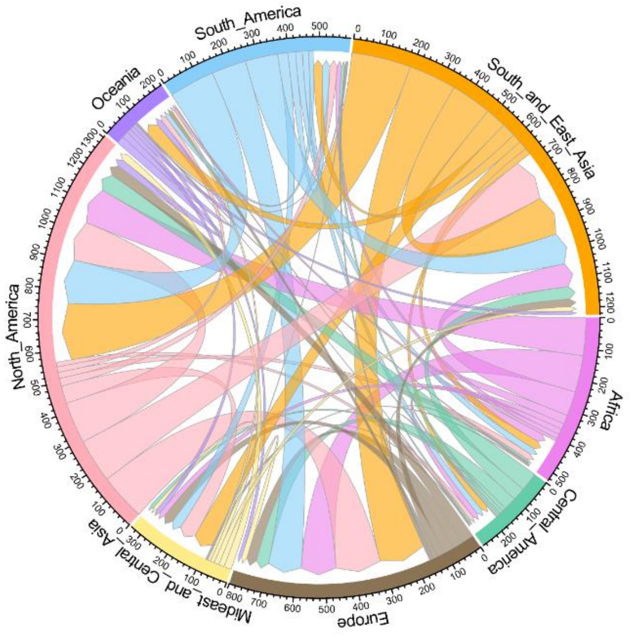

Fig S7. The global network of established traded alien vertebrates in term of species number for four groups. The figure is created by the dplyr, circlize and reshape2 packages in R (Network analysis in Supplementary Code 1). a, mammals; b, birds; c, reptiles; d, amphibians

a, mammals

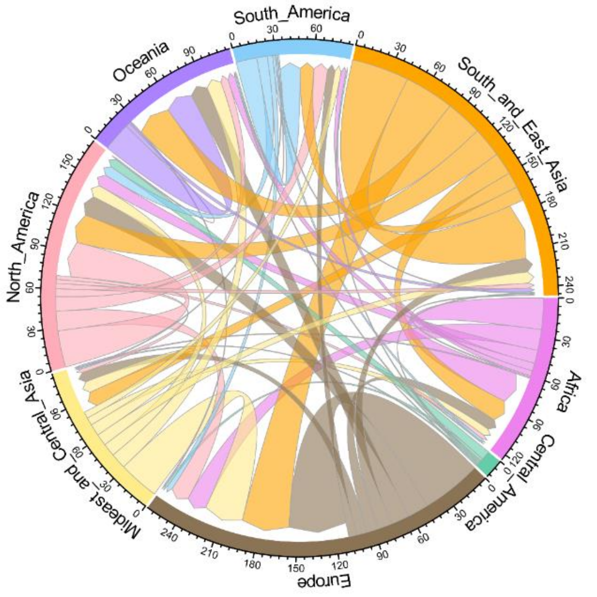

b, birds

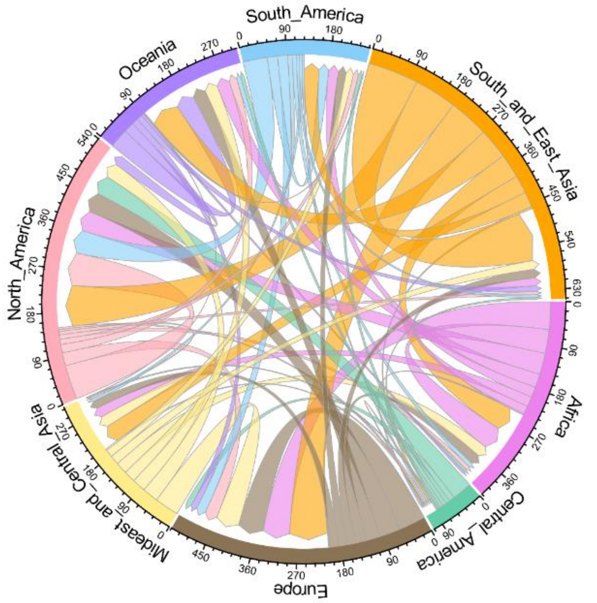

c, reptiles

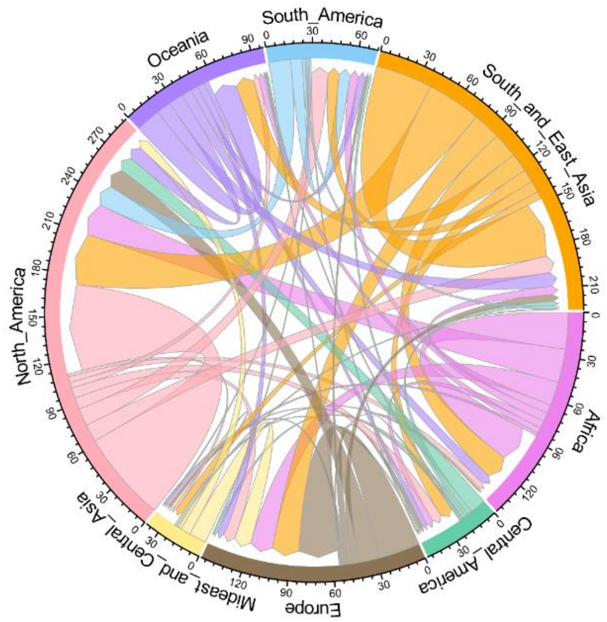

d, amphibians

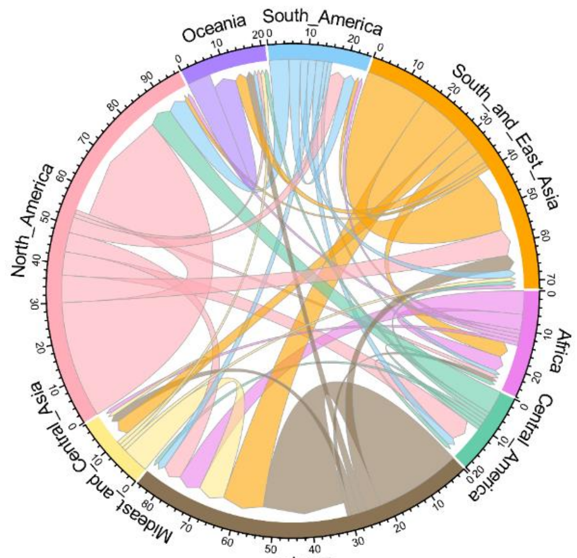

Table S1-11

Table S1. List of terrestrial vertebrates without data on geographic range on IUCN Red List web (https://www.iucnredlist.org/en, last visited on 30 April 2023)

Taxa	Scientific name	URL
Mammals	*Nycticeius aenobarbus*	https://www.iucnredlist.org/species/136562/4311281
Mammals	*Phoniscus aerosus*	https://www.iucnredlist.org/species/10967/21975373
Birds	*Caloenas maculata*	https://www.iucnredlist.org/species/22734732/95095848
Reptiles	*Hydrophis walli*	https://www.iucnredlist.org/species/176773/7301845
Reptiles	*Opisthotropis atra*	https://www.iucnredlist.org/species/199634/2608032
Reptiles	*Dipsas maxillaris*	https://www.iucnredlist.org/species/63772/12714506
Reptiles	*Hydrophis inornatus*	https://www.iucnredlist.org/species/176732/7292599
Reptiles	*Diploglossus microlepis*	https://www.iucnredlist.org/species/75169096/75169227
Reptiles	*Epictia signata*	https://www.iucnredlist.org/species/44582109/44582118
Reptiles	*Indotyphlops longissimus*	https://www.iucnredlist.org/species/118237195/118237205
Amphibians	*Caecilia mertensi*	https://www.iucnredlist.org/species/59520/11955115
Amphibians	*Ichthyophis humphreyi*	https://www.iucnredlist.org/species/59619/11969656
Amphibians	*Rhinella sima*	https://www.iucnredlist.org/species/54760/11185276
Amphibians	*Sphaenorhynchus platycephalus*	https://www.iucnredlist.org/species/56020/11410988

Table S2. Spearman rank correlation (*r* and probability) on the number of alien vertebrate and established alien species in trade across 193 countries among taxonomic groups. significant level (two sided), * $p \leqq 0.05$; ** $p \leqq 0.01$; *** $p \leqq 0.001$

Taxa	Birds	Reptiles	Amphibians
**Alien species**			
Mammals	.886**	.893**	.826**
Birds		.852**	.782**
Reptiles			.877**
**Established alien species**			
Mammals	.572**	.207**	.354**
Birds		.551**	.563**
Reptiles			.698**

Table S3. Summary of univariate linear mixed models for the relationships between alien vertebrate species number and each of socio-economic factors across upper middle or high-income countries. Each factor is log transformed to improve its linearity. Biogeographic realm is entered into a model as a random effect accounting for geographical autocorrelation. Estimate and p = standard estimate and probability of regression coefficient (two sided), respectively. N= number of countries with data available. R^2m=the variation explained by the fixed factors.

Factors	Estimate	n	t	p	R^2m
Population	0.399	100	11.473	<0.001	0.570
GDPpc	0.427	100	3.436	<0.001	0.071
Total value of import & export goods	0.461	85	18.192	<0.001	0.798

Table S4. Paired t-tests (two sided) on alien richness vs native richness in trade across 193 countries for each taxon.

Taxa	*n*	*t*	*P(two sided)*
Mammals	193	9.811	**<0.0001**
Birds	193	9.366	**<0.0001**
Reptiles	193	6.822	**<0.0001**
Amphibians	193	5.809	**<0.0001**

Table S5. The summary of univariate generalized linear mixed models (GLMMs) with a logit link and binomial error distribution, with establishment of species (establishment=1, unestablishment=0) as response variable and number of countries (log (1+x) transformed) or areas (km^2, log transformed)) involved in trade across alien species for each taxon. Order/family/genus enters into model as a nested random variable for accounting for taxonomic autocorrelation.

Taxa	Estimate	Pr(>|z|)
**Mammals**		
Intercept	-2.134	0.000
Country Number	0.527	0.000
Intercept	-4.332	0.000
Areas	0.368	0.002
**Birds**		
Intercept	-4.518	0.000
Country Number	1.669	0.000
Intercept	-7.961	0.000
Areas	0.681	0.000
**Reptiles**		
Intercept	-4.516	0.000
Country Number	1.773	0.000
Intercept	-8.462	0.000
Areas	0.768	0.000
**Amphibians**		
Intercept	-4.391	0.000
Country Number	1.036	0.003
Intercept	-3.226	0.011
Areas	-0.026	0.867

Table S6. Top models with △AICc<2 based on model selection for mammals. The full model is a linear mixed model with number of established traded alien species as the responsible variable and 9 factors as predictors (fixed effects) across 100 countries with up middle or high income. Biogeographical realms enter the models as a random factor. ΔAIC, the difference between each model and the highest ranked model; AICc, Akaike's information criterion adjusted for small sample sizes; Wi (Akaike weights), the probability that a model is best given the particular set of models considered; $R^2_{marginal}$, amount of variation that is explained by fixed factors (covariates); $R^2_{conditional}$, amount of variation that is explained by both fixed and random factors. Significance level (two sided): *, $p \leqq 0.05$; **, $p \leqq 0.01$; ***, $p \leqq 0.001$

Models	M1		M2		M3	
Parameters	Estimate	t value	Estimate	t value	Estimate	t value
Intercept	0.452	3.191**	0.534	3.712***	-0.096	-0.351
Area	-	-	-	-	-	-
Population density	-	-	-	-	-	-
GDPpc	-	-	-	-	-	-
Colonization pressure	0.393	7.755***	0.366	7.151***	0.336	5.704***
Insularity	0.157	2.599*	-	-	0.288	3.431***
Mean temperature	-0.030	-8.535***	-0.029	-8.049***	0.004	-7.693***
Mean precipitation	-	-	-	-	-	-
Congeneric richness	-	-	-	-	0.257	2.150*
Sampling effort	0.553	3.19**	0.606	3.423***	0.455	2.579*
AICc	28.26	-	28.75	-	28.83	-
Delta	0	-	0.48	-	0.56	-
Weight	0.24	-	0.19	-	0.18	-
R^2m	0.644	-	0.624	-	0.675	-
R^2c	0.726	-	0.720	-	0.710	-

Table S7. Top models with △AICc<2 based on model selection for birds. See Table S6 for details

Models	M1		M2	
Parameters	Estimate	t value	Estimate	t value
Intercept	-2.572	-6.528***	-3.23	-10.268***
Area	0.216	5.354***	0.303	12.615***
Population density	0.271	4.817***	0.368	8.541***
GDPpc	0.387	5.541***	0.506	9.106***
Colonization pressure	0.206	2.660*	-	-
Insularity	0.276	4.646***	0.306	5.045***
Mean temperature	-	-	-	-
Mean precipitation	-	-	-	-
Congeneric richness	-	-	-	-
Sampling effort	-	-	-	-
AICc	7.43	-	8.57	-
Delta	0	-	1.14	-
Weight	0.43	-	0.24	-
R^2m	0.598	-	0.609	-
R^2c	0.815	-	0.794	-

Table S8. Top models with △AICc<2 based on model selection for reptiles. See Table S6 for details

Models	M1		M2	
Parameters	Estimates	t value	Estimates	t value
Intercept	0.011	0.091	-0.028	-0.23
Area	-	-	-	-
Population density	-	-	-	-
GDPpc	-	-	-	-
Colonization pressure	0.336	7.545***	0.335	7.61***
Insularity	0.259	3.736***	0.238	3.407***
Mean temperature	-	-	-	-
Mean precipitation	-	-	-	-
Congeneric richness	-	-	-	-
Sampling effort	-	-	0.339	1.761
AICc	46.01	-	46.68	-
Delta	0	-	0.67	-
Weight	0.28	-	0.2	-
R^2m	0.297	-	0.315	-
R^2c	0.558	-	0.554	-

Table S9. Top models with △AICc<2 based on model selection for amphibians. See Table S6 for details

Amphibians	M1		M2		M3		M4	
Models	Estimate	t value	Estimate	t value	Estimate	t value	Estimate	t value
Intercept	0.099	1.227	-0.098	-0.932	0.193	2.337*	-2.758	-7.068***
Area	-	-	-	-	-	-	0.226	8.764***
Population density	-	-	-	-	-	-	0.275	6.009***
GDPpc	-	-	-	-	-	-	0.275	4.633***
Colonization pressure	0.264	8.188***	0.225	6.201***	0.248	7.543***	-	-
Insularity	0.170	2.801**	0.267	3.686***	-	-	0.185	2.887**
Mean temperature	-	-	-	-	-	-	-	-
Mean precipitation	-	-	-	-	-	-	0.171	2.583*
Congeneric richness	-	-	0.121	2.272*	-	-	-	-
Sampling effort	-	-	-	-	-	-	-	-
AICc	23.50	-	24.84	-	25.07	-	25.36	-
Delta	0	-	1.35	-	1.58	-	1.86	-
Weight	0.15	-	0.08	-	0.07	-	0.06	-
R^2m	0.338	-	0.390	-	0.289	-	0.34	-
R^2c	0.546	-	0.535	-	0.547	-	0.718	-

Table S10. Number of threatened species excluded from ISIS for different taxa.

Terms	Mammals	Birds	Reptiles	Amphibians	Total
Number of threatened species in ISIS Database	243	185	154	53	635
Number of threatened species in ISIS Database that have no records in other databases	48	37	22	20	127
Number of traded species from ISIS Database	690	2007	256	163	3116
Total number of traded species in GTVID	1247	3451	2278	804	7780
Proportion (%) of threatened species excluded from ISIS to total species in GTVID	3.85	1.07	0.97	2.49	1.63

Table S11. Proportions of different languages used by websites

Languages	No. of websites	Proportion
English	799	0.546
Russian	96	0.066
Spanish	80	0.055
French	76	0.052
German	59	0.040
Arabic	53	0.036
Czech	25	0.017
Portuguese	24	0.016
Dutch	21	0.014
Danish	17	0.012
Polish	17	0.012
Japanese	13	0.009
Swedish	12	0.008
Romanian	12	0.008
Azerbaijani	11	0.008
Turkish	10	0.007
English,Arabic	9	0.006
Latvian	9	0.006
Indonesian	9	0.006
Hungarian	8	0.005
Chinese	7	0.005
Hebrew	6	0.004
Estonian	6	0.004
Bulgarian	6	0.004
Italian	6	0.004
Finnish	6	0.004
Slovak	5	0.003
Ukrainian	5	0.003
English,Khmer	4	0.003
Croatian	4	0.003
Slovenian	4	0.003
Armenian	3	0.002
Greek, English	3	0.002
Korean	3	0.002
Vietnamese	3	0.002
Persian	3	0.002
Bosnian	3	0.002
Lithuanian	3	0.002
Norwegian	3	0.002

Latin	3	0.002
Arabic, English	2	0.001
Malay	2	0.001
English,Croatian	2	0.001
Georgian	2	0.001
Indonesian	1	0.001
English,Nepali	1	0.001
English,Chinese	1	0.001
Greek	1	0.001
French,Arabic	1	0.001
Burmese	1	0.001
Afrian Zulu	1	0.001
Boer	1	0.001
Ilokano	1	0.001
