# Supplementary material for: Quantifying global colonization pressures of alien vertebrates from wildlife trade
Source: Nat Commun. 2023 Nov 30;14:7914. doi: 10.1038/s41467-023-43754-6 (PMC10689770; doi:10.1038/s41467-023-43754-6)
Supplement: Supplementary file 3 — Description of Additional Supplementary Files [file 41467_2023_43754_MOESM3_ESM.pdf]

Description of Additional Supplementary Files

Supplementary Data 1. Families and number of species in family for trade species in GLVTD

Supplementary Data 2. Number of alien species in live wildlife trade across countries based on GLVTD

Supplementary Data 3. List of established trade species in GLVTD

Supplementary Data 4. Number of established alien species in live wildlife trade across countries based on GLVTD

Supplementary Data 5. Search phases in different languages used for websites or publications on the sale of mammals across 193 countries.

Supplementary Data 6. Search phases in different languages used for websites or publications on the sale of birds across 193 countries.

Supplementary Data 7. Search phases in different languages used for websites or publications on the sale of reptiles across 193 countries.

Supplementary Data 8. Search phases in different languages used for websites or publications on the sale of amphibians across 193 countries.

Supplementary Data 9. Publications used for retrieving data on historical online trade and physical stores.xlsx

Supplementary Data 10. References from the journal BioInvasions Records used for retrieving established terrestrial vertebrates and distributions.xlsx

Supplementary Code 1. R scripts used in this study
