# Supplementary material for: Quantifying global colonization pressures of alien vertebrates from wildlife trade
Source: Nat Commun. 2023 Nov 30;14:7914. doi: 10.1038/s41467-023-43754-6 (PMC10689770; doi:10.1038/s41467-023-43754-6)
Supplement: Supplementary file 14 — Supplementary Code 1 [file 41467_2023_43754_MOESM14_ESM.zip › Supplementary Code 1.docx]

**R scripts used in this study**

1. **Venn Diagram**

library(VennDiagram)

#*Four-set diagram, read data file, amphibians as an example

dat<-read.csv("F:\\Online trade-1\\NC-new analsis\\VN figures\\VN data\\VN amphibians.CSV",header = TRUE)

venn_list <- list(CITES = dat$CITES..116.species. ,

OTAPS = dat$OTAPS..577.species.,

LEMIS = dat$LEMIS..465.species.,

ISIS = dat$ISIS..163.species.)

names(venn_list)[1]<-"CITES(116)"

names(venn_list)[2]<-"OTAPS(577)"

names(venn_list)[3]<-"LEMIS(465)"

names(venn_list)[4]<-"ISIS(163)"

venn.diagram(venn_list,

filename = "F:\\Online trade-1\\NC-new analsis\\New analysis with LEMIS 1999-2022\\VN figures\\VN data\\VN-amphibians-new.png",

imagetype = 'png',

fill = c('red', 'blue', 'green', 'orange'),

alpha = 0.50,

cat.col = c('red', 'blue', 'green', 'orange'),

cat.cex = 0.9, cat.fontfamily = 'sans',

col = c('red', 'blue', 'green', 'orange'),

cex = 0.9, fontfamily = 'sans')

1. **Generalized linear mixed models (GLMMs)**

library(TMB)

library(glmmTMB)

# read data file

c<-read.csv("F:\\Online trade-1\\GLMM\\Amphibianarea.csv",header=TRUE)

ae<-glmmTMB(Invasion~+Countryarea+(1|Order/Family/Genus),data=c,family=binomial,na.action="na.pass")

summary(ae)

1. **Linear mixed models, model averaging and selection**

library(lme4)

library(MuMIn)

#read data, invasiveall=establishment richness, area=country area, pd=population density, bioregion=biogeographical realm

c<-read.csv("D:\\traded alien species\\LMM\\data2005.CSV",header = TRUE)

# perform linear mixed model

ae<-lmer(invasiveall~+area+pd+gdppc+island+temperature+rain++alien richness+unknown+congnereic richness+(1|bioregion),data=c,na.action="na.pass")

summary(ae)

#model averaging and selection

dd<-dredge(ae,beta="none",evaluate=TRUE,rank="AICc")

b <- get.models(dd,subset=TRUE)

avgb <- model.avg(b)

summary(avgb)

confint(avgb)

1. **Network analysis**

#fit the networks of species flows between native and alien countries in term of species number

Abbreviation

TheDataDirectory: file name. from: donor region, to: recipient region. num: number of trade alien species or established trade species.

.

library(dplyr)

library(circlize)

library(reshape2)

df <- read.csv("TheDataDirectory")

levels(df$from) = c("Africa","Central_America","Europe","Mideast_and_Central_Asia","North_America","Oceania","South_America","South_and_East_Asia")

levels(df$to) = c("Africa","Central_America","Europe","Mideast_and_Central_Asia","North_America","Oceania","South_America","South_and_East_Asia")

df = acast(df, from~to, value.var = 'value', sum)

#======================PLOT======================#

circos.clear()

circos.par(start.degree = 0,

gap.degree = .6, # +/- gap degree value adjusts node spacing & helps if labels overlap

track.margin = c(-0.1, 0.1),

points.overflow.warning = FALSE)

grid.col = c(Africa = "#EE82EE", Central_America = "#66CDAA", Europe = "#8B7355", Mideast_and_Central_Asia = "#FFEC8B",

North_America = "#FFAEB9", Oceania = "#AB82FF", South_America = "#87CEFA", South_and_East_Asia = "#FFA500")

chordDiagram(df, directional = 1,

direction.type = c("arrows", "diffHeight"), #settings here show direction of flow

diffHeight = -0.03,

transparency = 0.4, #helps see overlapping flows

grid.col = grid.col,

link.sort = TRUE,

link.arr.width = 0.05,

link.arr.type = 'big.arrow',

link.lwd = .5,

annotationTrack = c("grid", "axis"),

link.border = 'darkgrey',

self.link = 2,

big.gap = 3,

)

circos.trackPlotRegion(

track.index = 1,

bg.border = NA,

panel.fun = function(x, y) {

xlim = get.cell.meta.data("xlim")

sector.index = get.cell.meta.data("sector.index")

# Add names to the sector.

circos.text(

x = mean(xlim),

y = 2.9, #Change the height of names

labels = sector.index,

facing = "bending", #Change this setting for label placement

niceFacing = FALSE,

cex = 0.7

)

}

)
